# Total Adiponectin Is Inversely Associated with Platelet Activation and CHA_**2**_DS_**2**_-VASc Score in Anticoagulated Patients with Atrial Fibrillation

**DOI:** 10.1155/2014/908901

**Published:** 2014-02-26

**Authors:** Roberto Carnevale, Daniele Pastori, Mariangela Peruzzi, Elena De Falco, Isotta Chimenti, Giuseppe Biondi-Zoccai, Ernesto Greco, Antonino G. M. Marullo, Cristina Nocella, Francesco Violi, Pasquale Pignatelli, Camilla Calvieri, Giacomo Frati

**Affiliations:** ^1^Department of Internal Medicine and Medical Specialties, Sapienza University of Rome, 00161 Rome, Italy; ^2^Department of Medical-Surgical Sciences and Biotechnologies, Sapienza University of Rome, 04100 Latina, Italy; ^3^Department of Cardiovascular, Respiratory, Nephrological, Anesthesiological, and Geriatric Sciences, Policlinico Umberto, Sapienza University of Rome, 00161 Rome, Italy; ^4^Department of AngioCardioNeurology, IRCCS NeuroMed, 86077 Pozzilli, Italy

## Abstract

*Background*. Adiponectin (APN) possesses anti-inflammatory and antiatherogenic effects. Atrial fibrillation (AF) is burdened by enhanced systemic inflammation and platelet activation, as documented by increased blood levels of soluble CD40L (sCD40L). The interplay between APN and platelet activation in AF is still undefined. *Materials and Methods*. Circulating levels of APN and sCD40L were measured in 257 anticoagulated nonvalvular AF patients. Exclusion criteria were as follows: prosthetic heart valves, cardiac revascularization in the previous year, severe cognitive impairment, chronic infectious or autoimmune diseases, and active cancer. *Results*. Mean age was 72.9 (±8.7) years and 41.6% were female. Serum APN and plasmatic sCD40L were inversely correlated (*R* −0.626, *P* < 0.001). A progressive increase of sCD40L across tertiles of CHA_2_DS_2_-VASc score was observed (rS 0.473, *P* < 0.001), whilst APN was inversely correlated (rS −0.463,  *P* < 0.001). A multivariable linear regression analysis showed that CHA_2_DS_2_-VASc score (*B* −0.227, *P* < 0.001) and sCD40L (*B* −0.524, *P* < 0.001) correlated to APN. *Conclusions*. AF patients at high risk of stroke disclose low and high levels of APN and sCD40L, respectively, suggesting a role for APN if it favors platelet activation in vivo in this clinical setting. Enhancing APN levels may be a future goal to reduce the risk of vascular outcomes in AF patients.

## 1. Introduction

Atrial fibrillation (AF) is the most frequent supraventricular cardiac arrhythmia in the general population. Patients affected by AF, despite the recent introduction of novel oral anticoagulants, show an increased risk for ischemic vascular complications, such as ischemic stroke and cardiovascular mortality [1].

Several evidences suggest that AF is burdened by an enhanced systemic inflammatory status [2] and platelet activation [3, 4], as shown by the enhanced release of soluble CD40 ligand (sCD40L), which may affect AF-related thromboembolic events [5].

AF is characterized by the simultaneous presence of different atherosclerotic risk factors, frequently represented by arterial hypertension, diabetes, obesity, and dyslipidemia [6]. In particular, obesity is a well-known recognized risk factor for developing AF, and more recently weight loss has been associated with improved cardiac symptoms and reduced cardiac remodeling in AF patients [7, 8].

Adiponectin (APN) is the most abundant adipokine produced by adipose tissue, acting as an insulin-sensitizer and anti-inflammatory molecule [9, 10]. In addition to its metabolic properties, APN exerts antiatherogenic effects [11], and reduced serum APN levels have been found in patients with obesity, insulin resistance, and type 2 diabetes [12].

Data regarding APN levels in AF are controversial. Low serum APN levels have been found in paroxysmal AF patients compared to controls [13], whilst higher APN levels have been described in patients with permanent AF, compared to paroxysmal AF and controls [14]. Recently, Hernandez-Romero et al. have shown an association between low APN levels and cardiovascular outcomes in patients affected by AF [15]. Among the mechanisms accounting for such association, the interplay between APN and platelet activation could be considered as platelets play a key role in precipitating acute coronary and cerebrovascular disease [16]. Thus, APN is an antioxidant molecule, which inhibits platelet activation via lowering platelet oxidative stress [17, 18]. So far, data on APN interplay with platelet activation in AF patients are lacking. The aim of the study has been to examine the relationship between APN and platelet activation, as assessed by sCD40L, and to evaluate the association with CHA_2_DS_2_-VASc score in a cohort of anticoagulated nonvalvular AF patients.

## 2. Material and Methods

### 2.1. Study Design and Patient Selection

The study included 257 consecutive patients with AF who were referred to the Atherothrombosis Center of the Department of Internal Medicine and Medical Specialties of “Sapienza” University of Rome. All patients were treated with oral vitamin K antagonists according to CHA_2_DS_2_-VASc score [19] and the international normalized ratio was maintained in a therapeutic range of 2.0-3.0.

Exclusion criteria included presence of prosthetic heart valves, cardiac revascularization in the previous year, severe cognitive impairment (Alzheimer's disease and Parkinson's disease), chronic infectious diseases, autoimmune systemic diseases, and active cancer.

At enrollment, medical history, anthropometric data, and electrocardiogram were recorded and a sample of blood was collected from all patients. Arterial hypertension was defined as elevated blood pressure (≥140/≥90 mmHg) or taking antihypertensive therapy [20] regimen; diabetes was defined as a casual plasma glucose ≥200 mg/dL (11.1 mmol/L), fasting plasma glucose ≥126 mg/dL (7.0 mmol/L), or antidiabetic treatment [21]. Heart failure (HF) was defined as the presence of signs and symptoms typical of heart failure or reduced ejection fraction (EF ≤ 40%) [22]. Metabolic syndrome (MetS) was defined according to modified ATP-III criteria: abdominal obesity, given as waist circumference (in men >102 cm/>40, in women >88 cm/>35); triglycerides ≥150 mg/dL, HDL cholesterol (men <40 mg/dL, women <50 mg/dL); blood pressure ≥130/≥85 mmHg; fasting glucose ≥100 mg/dL [23].

All patients provided a written informed consent. The study protocol was approved by the local ethical board of “Sapienza” University of Rome and was carried out according to the principles of the Declaration of Helsinki [24].

### 2.2. Laboratory Analysis

Total serum APN levels were measured with a commercial immunoassay (Tema Ricerca, Italy) and expressed as ng/mL. Intra-assay and interassay coefficients of variation were 6% and 8%, respectively.

Platelet activation was assessed by the release of sCD40L. Blood samples were collected without stasis to minimize platelet activation from subjects who had fasted for at least 12 hours, directly mixed in a vacutainer (Vacutainer Systems, Belliver Industrial Estate) with 1 part of 3,8% Na citrate (ratio 9 : 1) and immediately centrifuged for 20 minutes at 2000 rpm at −4°C. Plasma samples were stored at −80°C until use.

Plasma levels of sCD40L were evaluated by immunoassay (Quantikine CD40 ligand, R&D Systems) and expressed as ng/mL. Intra-assay and interassay coefficients of variation were 6% and 7%, respectively.

### 2.3. Statistical Analysis

Categorical variables were reported as counts (percentages) and continuous variables as mean ± standard deviation (SD) or median and interquartile range (IQR) unless otherwise indicated. Independence of categorical variables was tested by *χ* ^2^ test. Normal distribution of parameters was assessed by Kolmogorov-Smirnov test. Student's unpaired *t*-test and Pearson product-moment correlation analysis were used for normally distributed continuous variables. After dividing population according to tertiles of CHA_2_DS_2_-VASc score, APN and sCD40L were analyzed. Group comparisons were performed using analysis of variance (ANOVA). Appropriate nonparametric tests (Mann-Whitney *U*-test, Kruskal-Wallis test, and Spearman rank correlation test [rS]) were employed for all the other variables. Only *P* values lower than 0.05 were considered as statistically significant. All tests were two-tailed and analyses were performed using computer software packages (SPSS-18.0, SPSS Inc.).

### 2.4. Sample Size

We calculated that 48 patients per group were required to have a 90% chance of detecting, as significant at the 5% level, a difference for APN levels between groups of 2 ng/mL with SD = 3 ng/mL.

## 3. Results

Baseline characteristics of all patients are reported in Table 1. Mean age was 72.9 (±8.7) years and 41.6% were female. One-hundred sixteen patients (45.1%) had paroxysmal AF, whilst 141 (54.9%) had persistent/permanent AF.

**Figure 1:**
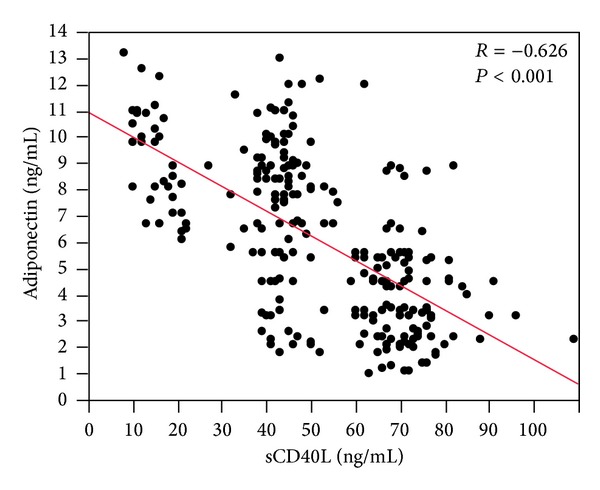
Linear regression analysis between sCD40L and adiponectin levels.

Median adiponectin value was 5.6 [3.2–8.6] ng/mL and median sCD40L was 49 [40–70] ng/mL. A significant inverse correlation between adiponectin and sCD40L was found (*R* −0.626, *P* < 0.001) (Figure 1).

**Figure 2:**
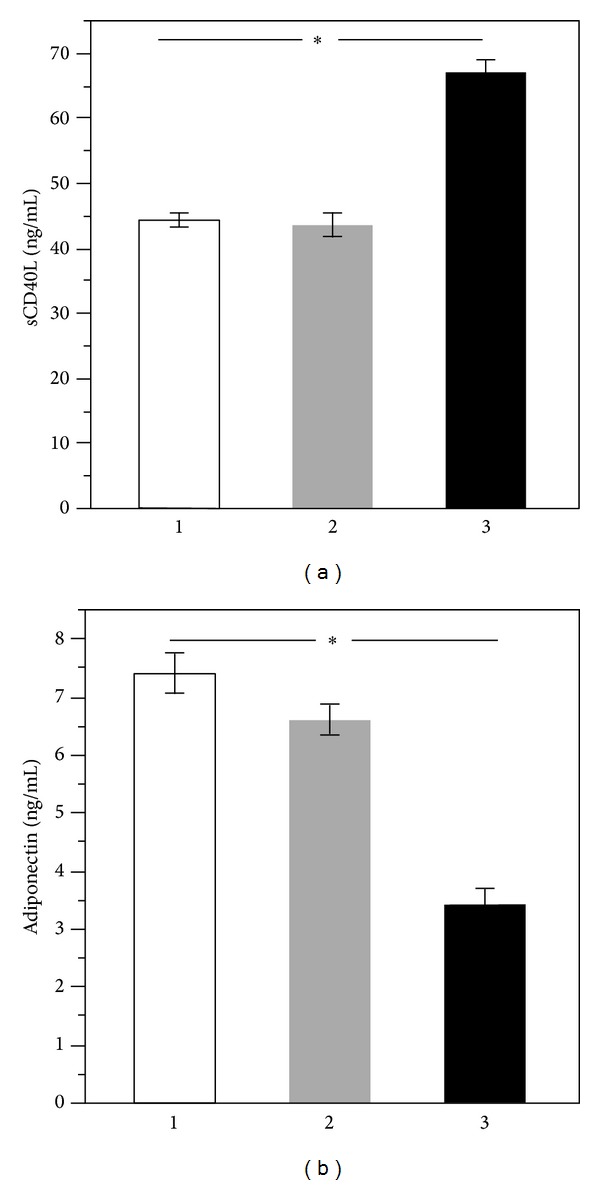
Median values of sCD40L (a) and adiponectin (b) according to tertiles of CHA_2_DS_2_-VASc score.

After dividing population according to tertiles of CHA_2_DS_2_-VASc score, a significant difference across tertiles was found both for sCD40L (*P* < 0.001) and APN (*P* < 0.001) (Table 2) (Figures 2(a) and 2(b)).

In particular, a progressive increase of sCD40L across tertiles of CHA_2_DS_2_-VASc score was observed (rS 0.473, *P* < 0.001) (Table 2). On the contrary, APN inversely correlated with tertiles of CHA_2_DS_2_-VASc score (rS −0.463, *P* < 0.001) (Table 2).

After adjustment for potential confounding factors, such as age, BMI, smoking habits, and diabetes, multivariable linear regression analysis showed that only CHA_2_DS_2_-VASc score (*R* ^2^ 0.431, *B* −0.227, *P* < 0.001) and sCD40L (*R* ^2^ 0.392, *B*  −0.524, *P* < 0.001) were independently correlated to adiponectin.

## 4. Discussion

The present study shows an inverse relationship between serum APN and CHA_2_DS_2_-VASc score, suggesting that lower antioxidant and higher inflammatory conditions are detectable in patients at a higher risk of stroke; the inverse relation between APN and sCD40L also suggests a role for oxidative stress in enhancing platelet activation in vivo.

Experimental and observational studies demonstrated that APN is directly involved in generating cardiovascular ischemic complications in the general population [25] and in different clinical settings at risk of vascular complications [15, 26–28]. Evidence derived from the in vitro study suggested that APN was able to inhibit the macrophage tissue factor, a key molecule promoting thrombus formation in disrupted plaques [29]. In APN-knockout mice, APN deficiency was associated with enhanced thrombus formation and platelet aggregation [11]. The interplay between APN and platelet activation has been explored in patients at risk of vascular complications [30, 31]. Thus, patients with MetS express receptors for APN on the platelet surface; platelet incubation with APN resulted in platelet aggregation inhibition and impaired CD40L release [31]. Similar results were observed in patients with type 2 diabetes [32], in which spontaneous platelet aggregation was inhibited by APN.

Clinical studies demonstrated that APN levels correlated with coronary artery disease severity [27], atherothrombotic and lacunar stroke types in men [28], and with incident heart failure in the physicians' health study [26]. Concerning AF, a recent study showed that APN levels were associated with cardiovascular events in anticoagulated AF female patients [15]. To investigate the mechanism potentially accounting for such inverse association, we analyzed the interplay between APN and sCD40L, which is a marker of platelet activation as it derives prevalently from CD40L released by the activated platelet [33, 34]. The inverse correlation between APN and sCD40L may provide novel insights on in vivo platelet activation in this setting as it suggests that the antioxidant status predisposes platelet activation. Such hypothesis is biologically plausible as previous studies consistently showed that oxidative stress is implicated in platelet activation via several mechanisms including formation of isoprostanes, which are chemically stable eicosanoids with proaggregating property [35], and an inactivation of nitric oxide, a powerful antioxidant molecule [36]. Also, platelet incubation with antioxidants different than APN similarly results in inhibition of platelet aggregation [37]. A previous study from our group [5] demonstrated that platelet activation, as assessed by circulating levels of sCD40L, is predictive of vascular outcomes in AF patients but the mechanism accounting for platelet activation was not explored. The present study provides insight into these findings suggesting that the antioxidant status may account for platelet activation in AF.

Another finding of the present study is the inverse correlation between APN and CHA_2_DS_2_-VASc score indicating that the risk of stroke is higher and the antioxidant status is lower. This finding is consistent with a previous study showing that serum levels of vitamin E, another molecule with antioxidant property, are inversely related to CHA_2_DS_2_-VASc score and predict cardiovascular outcomes in AF patients [38].

From our data it is, therefore, arguable that patients at a higher risk of stroke disclose a lower antioxidant status in association with platelet activation, both changes being potentially implicated in precipitating vascular outcomes by favoring atherosclerotic progression and thrombosis.

In conclusion, the present study shows that AF patients at high risk of stroke disclose low and high levels of APN and sCD40L, respectively, suggesting a role for APN in favoring platelet activation in vivo in this clinical setting. Enhancing APN levels may be a future goal to reduce the risk of vascular outcomes in AF patients.

## Figures and Tables

**Table 1:** Baseline characteristics.

Anthropometric and metabolic data	
Age (years)	72.9 ± 8.7
Female gender (%)	41.6
Body mass index (kg/m^2^)	27.0 ± 4.3
CHA_2_DS_2_-VASc score^#^	3 (2–4)
Adiponectin^#^ (ng/mL)	5.6 (3.2–8.6)
sCD40L^#^ (ng/mL)	49 (40–70)

Cardiovascular risk factors	

Hypertension (%)	88.3
Diabetes mellitus (%)	17.9
Heart failure (%)	16.7
History of stroke/TIA (%)	14.0
History of MI (%)	23.0
Metabolic syndrome (%)	51.0

Concomitant therapies	

(i) Antiplatelets (%)	11.3
(ii) ACE inhibitor/ARBs (%)	68.1
(iii) *β* blockers (%)	45.1
(iv) Calcium channel blockers (%)	33.9
(v) Statins (%)	45.5
(vi) Antiarrhythmic drugs (%)	34.2

^#^Data are expressed as median and interquartile range.

sCD40L: soluble CD40 ligand, TIA: transient ischemic attack, MI: myocardial infarction, ACE: angiotensin converting enzyme, and ARBs: angiotensin receptor blockers.

**Table 2:** Median values of adiponectin and sCD40L according to tertiles of CHA_2_DS_2_-VASc score.

	CHA_2_DS_2_-VASc score			*P* value
	1st tertile	2nd tertile	3rd tertile	
Adiponectin	8.4 (5.5–9.2)	6.5 (4.5–8.7)	3.1 (2.3–4.3)	**<0.001**
sCD40L	44 (40.0–47.5)	45 (22–68)	70 (67–74)	**<0.001**

sCD40L: soluble CD40 ligand.
